# The ratio of red light to far red light alters *Arabidopsis* axillary bud growth and abscisic acid signalling before stem auxin changes

**DOI:** 10.1093/jxb/erw479

**Published:** 2017-01-06

**Authors:** Srinidhi V. Holalu, Scott A. Finlayson

**Affiliations:** 1 Department of Soil and Crop Sciences, Texas A&M University, College Station, TX 77843,USA; 2 Faculty of Molecular and Environmental Plant Sciences, Texas A&M University, College Station, TX 77843,USA

**Keywords:** Abscisic acid, Arabidopsis, auxin, axillary bud, branch, competition, phytochrome, R:FR.

## Abstract

*Arabidopsis thaliana* shoot branching is inhibited by a low red light to far red light ratio (R:FR, an indicator of competition), and by loss of phytochrome B function. Prior studies have shown that phytochrome B deficiency suppresses bud growth by elevating systemic auxin signalling, and that increasing the R:FR promotes the growth of buds suppressed by low R:FR by inhibiting bud abscisic acid (ABA) accumulation and signalling. Here, systemic auxin signalling and bud ABA signalling were examined in the context of rapid bud responses to an increased R:FR. Increasing the R:FR promoted the growth of buds inhibited by a low R:FR within 6 h. Relative to a low R:FR, bud ABA accumulation and signalling in plants given a high R:FR showed a sustained decline within 3 h, prior to increased growth. Main stem auxin levels and signalling showed a weak, transient response. Systemic effects and those localised to the bud were further examined by decapitating plants maintained either under a low R:FR or provided with a high R:FR. Increasing the R:FR promoted bud growth before decapitation, but decapitated plants eventually formed longer branches. The data suggest that rapid responses to an increased R:FR may be mediated by changes in bud ABA physiology, although systemic auxin signalling is necessary for sustained bud repression under a low R:FR.

## Highlight

Increasing the ratio of red to far red light promotes axillary bud outgrowth and suppresses bud abscisic acid signalling within 3 h, before stem auxin signalling is affected.

## Introduction

Shoot architecture is determined to a large extent by the growth and development of branches, which is usually a plastic trait regulated by genetics, the environment, and interactions between the two. Axillary buds formed in the leaf axils from axillary meristems can remain arrested or elongate into branches of variable sizes, generating a wide variety of plant forms. Branching confers adaptation to diverse ecological conditions and contributes to fitness. In crops, branching impacts yield and productivity, and thus has been an important trait in domestication and is often targeted by breeders when developing novel cultivars/varieties.

In Arabidopsis (*Arabidopsis thaliana*), axillary buds may remain small in a quasi-dormant state, or elongate and form a branch. The transition from quasi- dormancy to sustained growth is determined by many factors intrinsic and external to the bud (Waldie *et al.*, 2010; Domagalska and Leyser, 2011; Janssen 2014; Rameau *et al.*, 2015). In typical Arabidopsis accessions grown under long days, the buds formed in the upper leaf axils begin to elongate first, whereas lower buds show a sequential delay of elongation (Hempel and Feldman, 1994). The growth of lower buds is variable and is contextually controlled by developmentally derived signals, environmental factors, or the combined action of both (Finlayson, 2007; Finlayson *et al.*, 2010; Reddy *et al.*, 2013; Reddy and Finlayson, 2014; Reddy *et al.*, 2014). In many species of both monocots and eudicots, light signals that indicate a competitive or shaded environment inhibit branching (Deregibus *et al.*, 1983; Davis and Simmons, 1994; Robin *et al.*, 1994; Donohue and Schmitt, 1999; Wan and Sosebee, 1998; Kebrom *et al.*, 2006; Finlayson *et al.*, 2010; Kebrom *et al.*, 2010). The low red light to far red light ratio (R:FR) generated in these competitive and/or shaded environments is sensed by the phytochrome (phy) family of photoreceptors, including the major R:FR sensor phyB. Signals perceived by phyB evoke a suite of adaptive responses termed the shade avoidance syndrome (SAS), including reduced branching (Casal, 2012). Several studies have shown that the abundance of the natural auxin indole-3-acetic acid (IAA) increases rapidly in young Arabidopsis seedlings in response to a low R:FR and contributes to the shade avoidance syndrome (Tao *et al.*, 2008, Hornitschek *et al.*, 2012, Pacín *et al.*, 2016). A low R:FR and phyB deficiency have been shown to inhibit branching in Arabidopsis by altering the expression of a variety of genes and pathways that operate both systemically and in a bud autonomous fashion (Finlayson *et al.*, 2010, González-Grandío *et al.*, 2013, Reddy *et al.*, 2013, Reddy and Finlayson, 2014).

Hormonal pathways regulating axillary bud growth and branching have received considerable attention. Auxin has been implicated as a systemic regulator of branching. Auxin synthesized in the main shoot apex and upper branches is transported basipetally in the polar auxin transport stream and inhibits bud growth indirectly, without entering the bud. The inhibitory influence of superior shoots on the development of lower branches is a form of correlative inhibition known as apical dominance. The correlative inhibition of lower bud growth can be attributed to the inhibitory effects of auxin sourced from more apical organs, though other mechanisms may also be involved (Cline, 1997; Morris *et al.*, 2005). The depletion of auxin (and possibly other factors) in the main shoot, either by decapitation or by impeding auxin transport with chemical inhibitors, can result in robust promotion of bud growth. The precise mechanism by which auxin exerts its indirect effects on bud growth remains unresolved although interesting models have been presented. One model proposes that auxin impacts secondary messengers (e.g. cytokinins and strigolactones) that move into the bud to promote or inhibit growth (Brewer *et al.*, 2009; Dun *et al.*, 2009; Brewer *et al.*, 2015). Another model provides evidence that axillary buds and superior apices compete for auxin transport capacity in the main stem (Bennett *et al.*, 2006; Prusinkiewicz *et al.*, 2009; Balla *et al.*, 2011; Bennett *et al.*, 2016). Axillary buds grow only if they are capable of establishing an auxin efflux into the main shoot polar auxin transport stream. phyB-deficient Arabidopsis exhibits a constitutive shade avoidance syndrome that includes exaggerated apical dominance. This response was attributed to elevated auxin signalling in the main stem, independent of auxin abundance in this tissue (Reddy and Finlayson, 2014).

Many studies have associated elevated bud ABA abundance with the inhibition of branching (Tamas *et al.*, 1979; Knox and Wareing, 1984; Gocal *et al.*, 1991; Mader *et al.*, 2003), including in the context of responses to the R:FR (Tucker and Mansfield, 1972; Tucker, 1977). Pharmaceutical approaches have shown that exogenous ABA treatment inhibits branching in a variety of species (Arney and Mitchell, 1969; Chatfield *et al.*, 2000; Cline and Oh, 2006), whereas the ABA biosynthesis inhibitor fluridone promoted branching in rose (*Rosa hybrida*) (Le Bris *et al.*, 1999). Likewise, *in vitro* explants of genetically modified Poplar (*Populus* X *canescens* [Ait.] Sm.) with reduced ABA sensitivity exhibited enhanced branching (Arend *et al.*, 2009). Bud growth in sugarcane (*Saccharum officinarum*) has been associated with reduced bud ABA abundance and modification of ABA signalling by small RNAs (Ortiz-Morea *et al.*, 2013). Buds of sorghum (*Sorghum bicolor*) deficient in phyB exhibited retarded growth relative to wild type, and overexpressed a set of ABA-related genes (Kebrom and Mullet, 2016). Exposing Arabidopsis grown under a high R:FR to a low R:FR suppressed branching and enhanced ABA signalling (González-Grandío *et al.*, 2013). The opposite approach of growing Arabidopsis under a low R:FR inhibited the growth of specific lower buds, which could then be rapidly promoted to grow by increasing the R:FR (Reddy *et al.*, 2013). ABA signalling in these buds was suppressed by the increased R:FR and bud ABA levels declined within 12 h. A role for ABA as a regulator of branching was demonstrated using mutants deficient in ABA biosynthesis that exhibited incomplete suppression of bud growth in a low R:FR (Reddy *et al.*, 2013). ABA has now been shown to inhibit lower bud growth under both high and low R:FRs and may act in part by suppressing the expression of genes associated with the cell cycle (Yao and Finlayson, 2015). ABA also inhibited the expression of genes associated with the bud autonomous auxin pathway and inhibited the accumulation of IAA in the bud, which may be associated with the establishment of bud auxin efflux necessary for growth (Yao and Finlayson, 2015).

Various pathways regulating branch development, including auxin, strigolactones, cytokinins and sugars, have been shown to be integrated by the TEOSINTE BRANCHED1/CYCLOIDEA/PCF (TCP) domain transcription factor BRANCHED 1 (BRC1), or its homologs in other species (Aguilar-Martínez *et al.*, 2007; Finlayson, 2007; Braun *et al.*, 2012; Dun *et al.*, 2012; Chen *et al.*, 2013; Mason *et al.*, 2014; Barbier *et al.*, 2015). ABA is an exception, because it functions downstream of BRC1 (Yao and Finlayson, 2015). BRC1 may not only target ABA because it has also been shown to regulate the expression of a variety of cell cycle- and ribosome-related genes not previously identified as ABA responsive (González-Grandío *et al.*, 2013).

Although roles for systemic auxin signalling in the main stem and for bud-localized ABA have been shown to contribute to axillary bud responses to competition signals mediated by phyB, much still remains unknown. The temporal kinetics of bud growth modification by the R:FR have not yet been determined. If the effects of the R:FR on bud development are transduced mainly by bud-localized mechanisms then a more rapid response may be anticipated, whereas systemic effects may require more time to manifest. Furthermore, the relative contributions of the systemic auxin and bud-localized ABA pathways have not been determined and nor have the potential interactions between the two. In the present study the timing of the bud growth response to an increased R:FR was defined, as were changes in the physiology associated with the two targeted hormone pathways. It was hypothesized that increasing the R:FR would promote bud growth within hours and that ABA and auxin pathways would exhibit altered behaviour prior to changes in bud growth. It was also hypothesized that an increased R:FR would alter bud-localized ABA homeostasis and signalling prior to changes in auxin signalling in the main stem.

## Materials and methods

### Plant growth, treatments, and bud elongation measurements

Plant growth and light conditions were as described in Reddy *et al.* (2013). Wild-type *A. thaliana* (Col-0, ABRC CS60000) was used throughout. Seeds were stratified at 4°C for 3 days in the dark, and then sown on Sunshine LC1 soil-less media in 50 mL conical tubes that were cut down to 30 mL. The plants were grown in a growth chamber modified with an overhead array of FR light-emitting diodes (735 nm). The chamber was split into two equal parts with a light-impermeable baffle. Photosynthetically active radiation was provided by fluorescent lamps at 180 μmoles·m^−2^·s^−1^ photosynthetic photon flux density. Plants were initially exposed to a high R:FR (3.5) for 1 day, and then given supplemental FR to reduce the R:FR to 0.09. Spectra of the light sources are provided in Supplementary Fig. 1 (available at *JXB* online). Plants received a photoperiod of 16/8 h light/dark and temperatures of 24/18°C day/night. At 3 days after anthesis, the FR light-emitting diodes were turned off 1 h after dawn to increase the R:FR to 3.5 in one side of the chamber without changing the photosynthetic photon flux density.

For bud elongation measurements, plants were matched for uniformity of rosette and cauline leaf numbers, maturity, height, and bud size. Decapitation was conducted by cutting the main stem approximately 5 mm above the rosette, but always below the lowest cauline leaf. Bud n-2 (the third bud from the top of the rosette) was imaged with a digital camera equipped with a macro lens at various times after initiating light and/or decapitation treatments. ImageJ software was used to process the images to determine bud lengths.

### Analysis of ABA and IAA abundance

Bud n-2 and basal main stem sections (15 mm adjacent to the rosette) were harvested into liquid N_2_ at 0, 1, 3, 6 and 12 h after increasing the R:FR for ABA and IAA analyses. Masses were determined immediately after harvest. ABA and IAA were extracted and measured using isotope dilution selected ion monitoring gas chromatography-mass spectroscopy as described previously (Yao and Finlayson, 2015). Each measurement was derived from four biological replicates composed of approximately 15 buds or six stem segments.

### Gene expression analysis

Bud n-2 and basal main stem sections were harvested at the times indicated after increasing the R:FR as described above. Total RNA was extracted, cDNA was synthesized, and quantitative PCR was conducted as previously described (Su *et al.*, 2011), except that expression of *UBC21* was used for normalization. Hormone-responsive genes for the quantification of ABA and IAA signal outputs were identified from the stringent set described by Goda *et al.* (2008). Specific gene targets were selected based on expression response to the appropriate stimulus (ABA or auxin) and demonstrated expression in buds (from Reddy *et al.* 2013). To determine the average signalling status, gene expression values were normalized to the mean expression of a particular gene, and this value was then averaged with others representing the same pathway. Expression values for genes exhibiting repressed expression in response to hormones were inverted before normalizing and averaging. Primers for *BRC1* were taken from Aguilar-Martínez *et al.* (2007). Primers for *INDOLE-3-ACETIC ACID INDUCIBLE 19* (*IAA19*) and *GH3.5* were obtained from Effendi *et al.* (2011). Primers for *PROLIFERATING CELL NUCLEAR ANTIGEN 1* (*PCNA1*) and *TRYPTOPHAN AMINOTRANSFERASE OF ARABIDOPSIS 1* (*TAA1*) are given in Finlayson *et al.* (2010). Primer sequences for *IAA2*, *IAA3*, *IAA6*, and *IAA29* are given in Reddy and Finlayson (2014). Primer sequences for *HISTONE H1-3* (*HIS1-3*) are provided in Su *et al.* (2011). Primer sequences for *CYCLIN A2;1* (*CYCA2;1*) and *PIN-FORMED 1* (*PIN1*) are given in Yao and Finlayson (2015). The sequences of other primers used are specified in Table 1. Each measurement was derived from four biological replicates composed of approximately 10 buds or six stem segments.

**Table 1.** Sequences of primers used for quantitative PCR

Target	Forward primer (5′ to 3′)	Reverse primer (5′ to 3′)
*UBC21* (At5G25760)	CTGCGACTCAGGGAATCTTCTAA	TTGTGCCATTGAATTGAACCC
*NAP* (At1g69490)	CGTCTCCATGATTCACGTAAAGCA	TACTTCGTCCATGAAACCCTCTTG
*RAP2.6* (At1g43160)	TGGACGATGGGTCATAAGAGAGAA	CTCCAAGGACATTGAGCTTTCACA
*PP2-A5* (At1g65390)	GAGATCTTTCCATTGCATGGTCAG	TACCTTGTCCTCGGGGTCAAATAT

### Statistics

Statistical significance was determined by Student’s *t*-test (two-tailed), or ANOVA followed by Tukey’s honest significant difference test using R. Significance was determined at α = 0.05.

## Results

### Bud growth was promoted rapidly in response to an increased R:FR

**Fig. 1.**
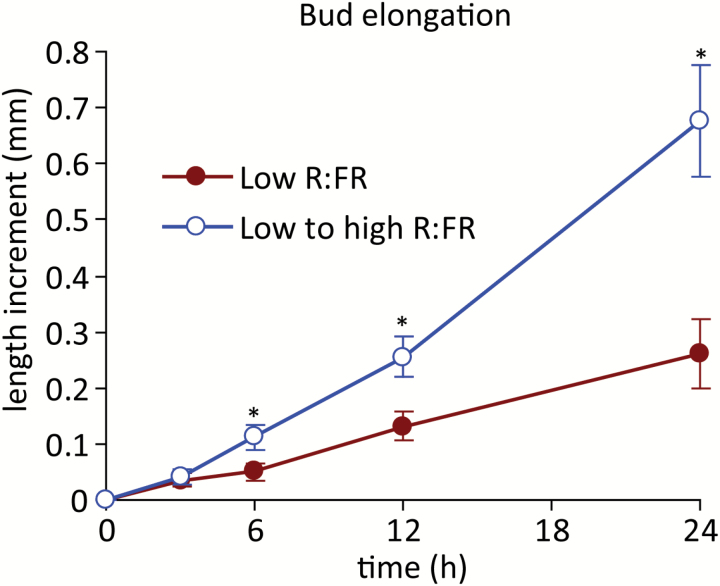
Elongation of bud n-2 under a low R:FR and at various times after increasing the R:FR. Data are means ± SE with *n* = 13. Asterisks indicate a significant difference between a low and low to high R:FR at α = 0.05. This figure is available in colour at *JXB* online.

The R:FR was previously shown to regulate the growth of buds at specific rosette positions, with bud n-2 (the third bud from the top of the rosette) showing strong repression by low R:FR (Reddy *et al.*, 2013). This prior work showed that the growth of bud n-2 was promoted within 24 h of increasing the R:FR. To further define the process, an experiment was conducted to determine the kinetics of the bud elongation response to the R:FR with greater temporal resolution than in the previous study. Plants were grown under a low R:FR to suppress bud elongation and, at 3 days after anthesis, one half of the plants were provided with a high R:FR (beginning 1 h after dawn) to promote the growth of bud n-2. The buds were not significantly different in size at the start of the experiment (low R:FR = 1.76 ± 0.10 mm, low to high R:FR = 1.69 ± 0.08 mm). An increased R:FR stimulated elongation within 6 h of initiation of the treatment, and by 24 h the bud growth increment of plants given a high R:FR was more than double that of plants maintained under a low R:FR (Fig. 1).

### *BRC1* expression was rapidly suppressed in response to increased R:FR

**Fig. 2.**
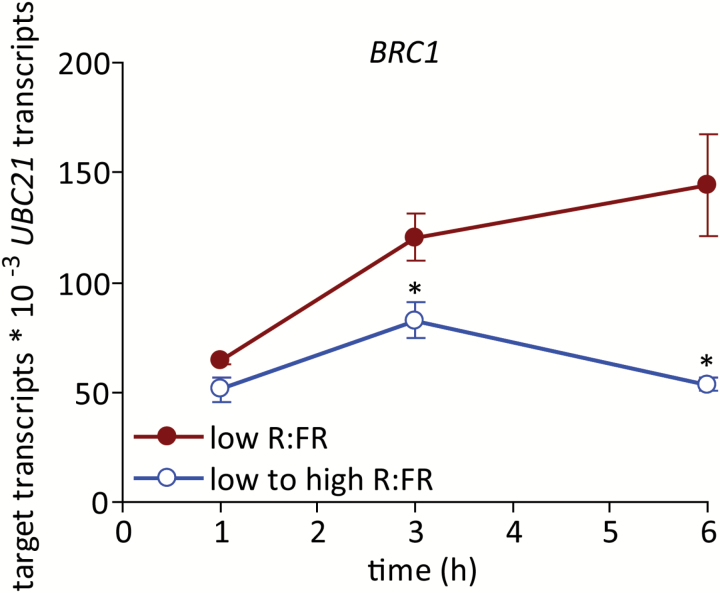
Expression of *BRC1* in bud n-2 under a low R:FR and at various times after increasing the R:FR. Data are means ± SE with *n* = 4. Asterisks indicate a significant difference between a low and low to high R:FR at α = 0.05. This figure is available in colour at *JXB* online.

Relative to a low R:FR, expression of the bud growth inhibitor *BRC1* was suppressed within 3 h of increasing the R:FR and declined to less than one half of the level in plants maintained under a low R:FR by 6 h (Fig. 2). Expression of *BRC1* was dynamic even in buds of plants maintained under a low R:FR, with abundance displaying a steady increase over the duration of the experiment. This molecular probe of bud status thus indicates that a high R:FR alters the bud’s physiology within 3 h, before the measured increase in elongation.

### ABA abundance and ABA signalling in buds declined in response to an increased R:FR prior to measured effects on bud elongation

**Fig. 3.**
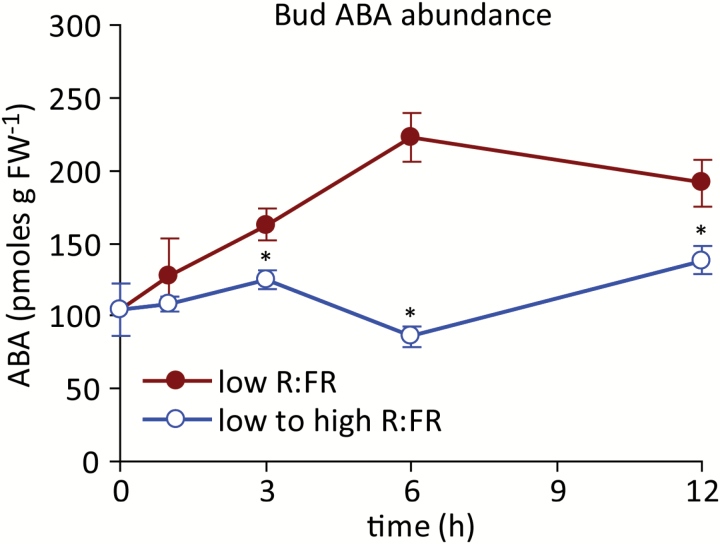
ABA abundance in bud n-2 under a low R:FR and at various times after increasing the R:FR. Data are means ± SE with *n* = 4. Asterisks indicate a significant difference between a low and low to high R:FR at α = 0.05. This figure is available in colour at *JXB* online.

**Fig. 4.**
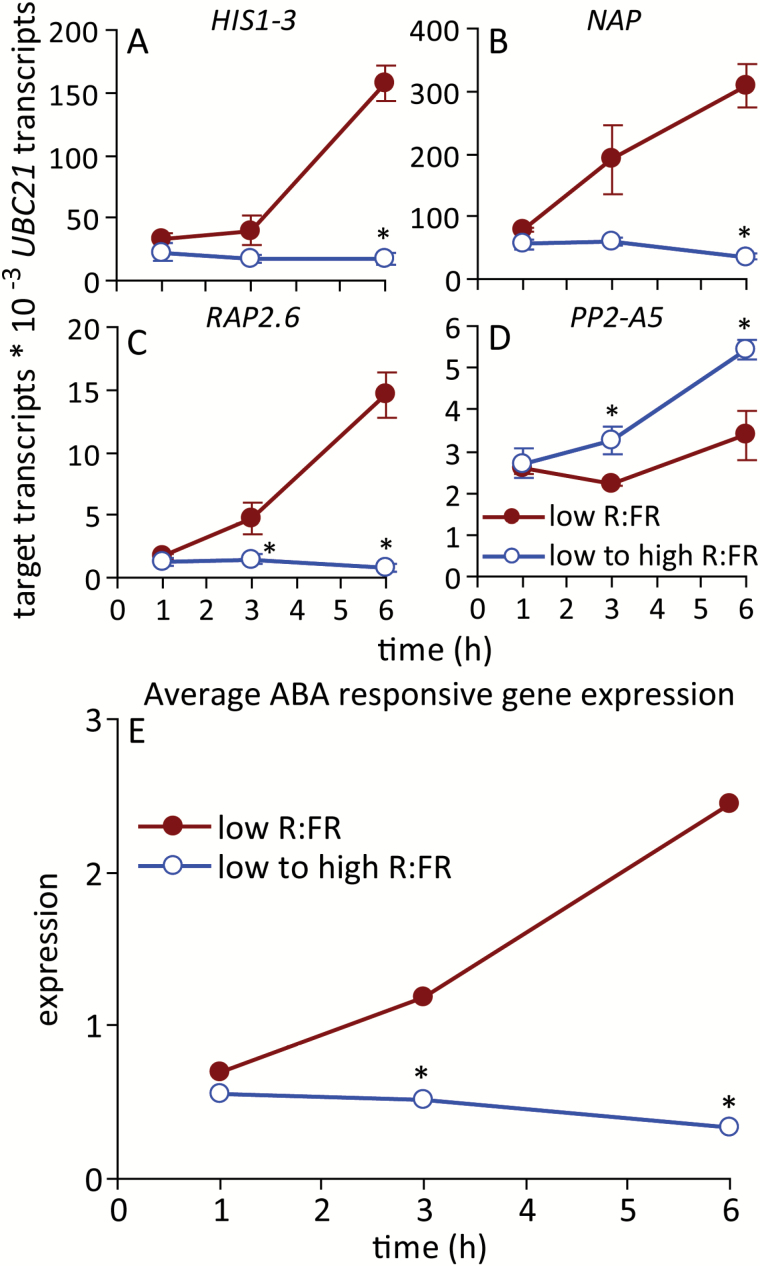
Expression of ABA-responsive genes in bud n-2 under a low R:FR and at various times after increasing the R:FR. (**A**) *HIS1-3*, (**B**) *NAP*, (**C**) *RAP2.6*, (**D**) *PP2-A5*, (**E**) average positive normalized expression. Data are means ± SE with *n* = 4. Asterisks indicate a significant difference between a low and low to high R:FR at α = 0.05. This figure is available in colour at *JXB* online.

Bud ABA levels were previously shown to decline within 12 h of initiating the high R:FR treatment (Reddy *et al.*, 2013). In the present study, ABA levels increased steadily to 6 h in buds of plants maintained under a low R:FR, then declined slightly (Fig. 3). Exposure to a high R:FR reduced bud ABA abundance relative to low R:FR within 3 h, and this reduction was maintained throughout the time course. A panel of ABA-responsive genes (Goda *et al.*, 2008) was then surveyed for expression responses to provide a readout of ABA signalling status. The expression of ABA-induced genes (*HIS1-3*, *ARABIDOPSIS NAC DOMAIN CONTAINING PROTEIN 29* [*NAP*] and *RELATED TO AP2 6* [*RAP2.6*]), increased significantly up to 6 h in a low R:FR, whereas expression of the ABA-repressed gene *PHLOEM PROTEIN 2 A5* (*PP2-A5*) was more stable (Fig. 4). An increased R:FR suppressed the expression of *RAP2.6* within 3 h and of *HIS1-3* and *NAP* within 6 h. *PP2-A5* abundance was increased within 3 h. The abundance of each gene was normalized to the mean, the expression of *PP2-A5* was inverted, and the values were averaged to provide an overall indicator of ABA signalling status (Fig. 4). Overall, bud ABA signalling was significantly suppressed by 3 h after increasing the R:FR.

### Cell cycle- and auxin-related outputs of bud ABA signalling were altered in response to an increased R:FR

**Fig. 5.**
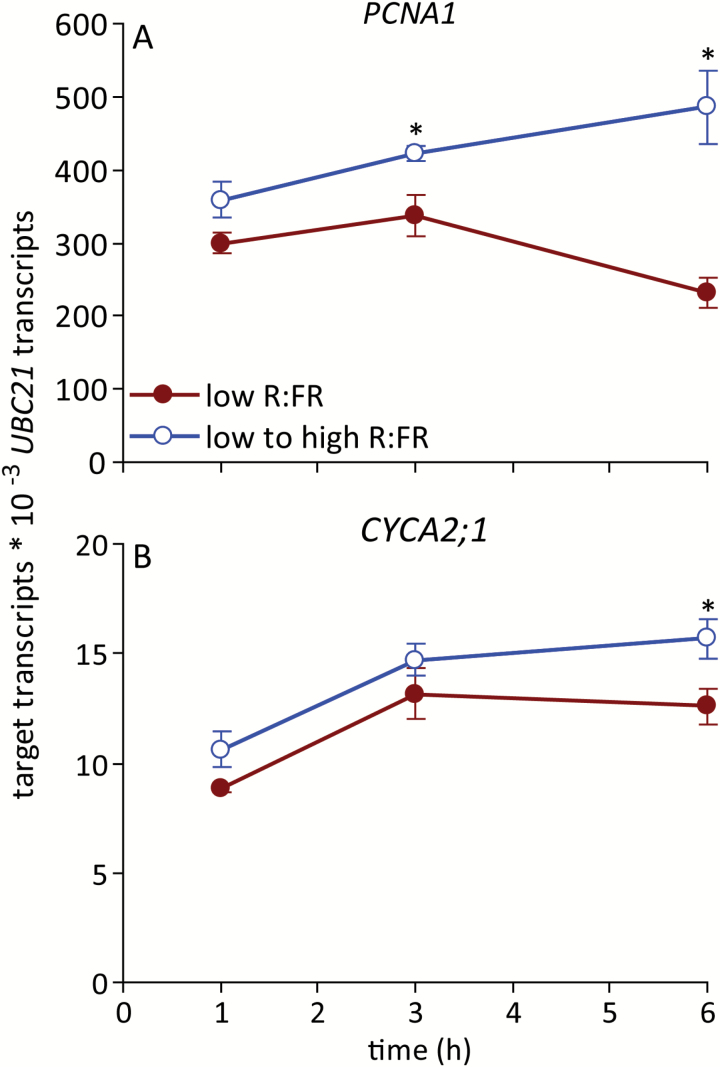
Expression of (**A**) *PCNA1* and (**B**) *CYCA2;1* in bud n-2 under a low R:FR and at various times after increasing the R:FR. Data are means ± SE with *n* = 4. Asterisks indicate a significant difference between a low and low to high R:FR at α = 0.05. This figure is available in colour at *JXB* online.

*PCNA1* encodes a processivity factor necessary for the cell cycle that may also act as a regulatory component of the process (Strzalka and Ziemienowicz, 2011; Koundrioukoff *et al.*, 2000). Bud *PCNA1* expression was previously shown to be repressed by ABA (Yao and Finlayson, 2015). *PCNA1* expression showed variation over the time course, declining at the 6 h time point in a low R:FR (Fig. 5). Like *BRC1*, *PCNA1* expression was rapidly altered in response to an increased R:FR, increasing significantly relative to low R:FR by 3 h. Prior research indicated that expression of the cell cycle regulator *CYCA2;1* may also be inhibited by ABA (Yao and Finlayson, 2015). Like *PCNA1*, *CYCA2;1* expression was also promoted by an increased R:FR, but the effect was apparent slightly later, at 6 h (Fig. 5).

**Fig. 6.**
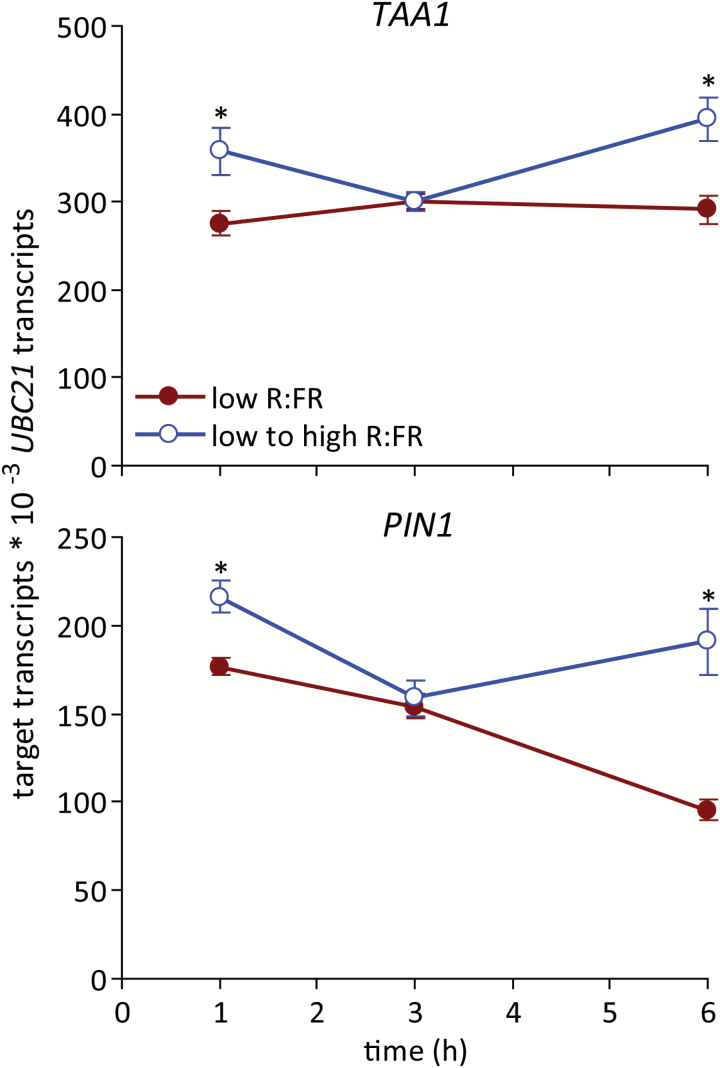
Expression of (**A**) *TAA1* and (**B**) *PIN1* in bud n-2 under a low R:FR and at various times after increasing the R:FR. Data are means ± SE with *n* = 4. Asterisks indicate a significant difference between a low and low to high R:FR at α = 0.05. This figure is available in colour at *JXB* online.

Bud autonomous expression of auxin biosynthesis and transport genes has been associated with ABA regulation of bud fate (Yao and Finlayson, 2015). The auxin biosynthesis gene *TAA1* and the auxin transporter gene *PIN1* showed similar expression patterns, with elevated abundance at 1 and 6 h after increasing the R:FR (Fig. 6). However, both genes showed equivalent accumulation under the different light treatments at 3 h.

The expression patterns of these genes are generally consistent with their potential functions regulating bud development downstream of ABA.

### Stem IAA abundance declined transiently in response to an increased R:FR, but auxin signalling showed little effect

**Fig. 7.**
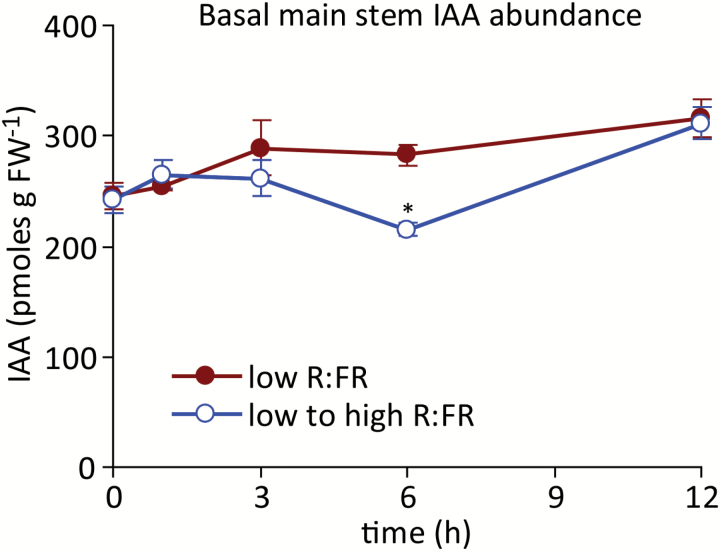
IAA abundance in basal main stem segments under a low R:FR and at various times after increasing the R:FR. Data are means ± SE with *n* = 4. Asterisks indicate a significant difference between a low and low to high R:FR at α = 0.05. This figure is available in colour at *JXB* online.

**Fig. 8.**
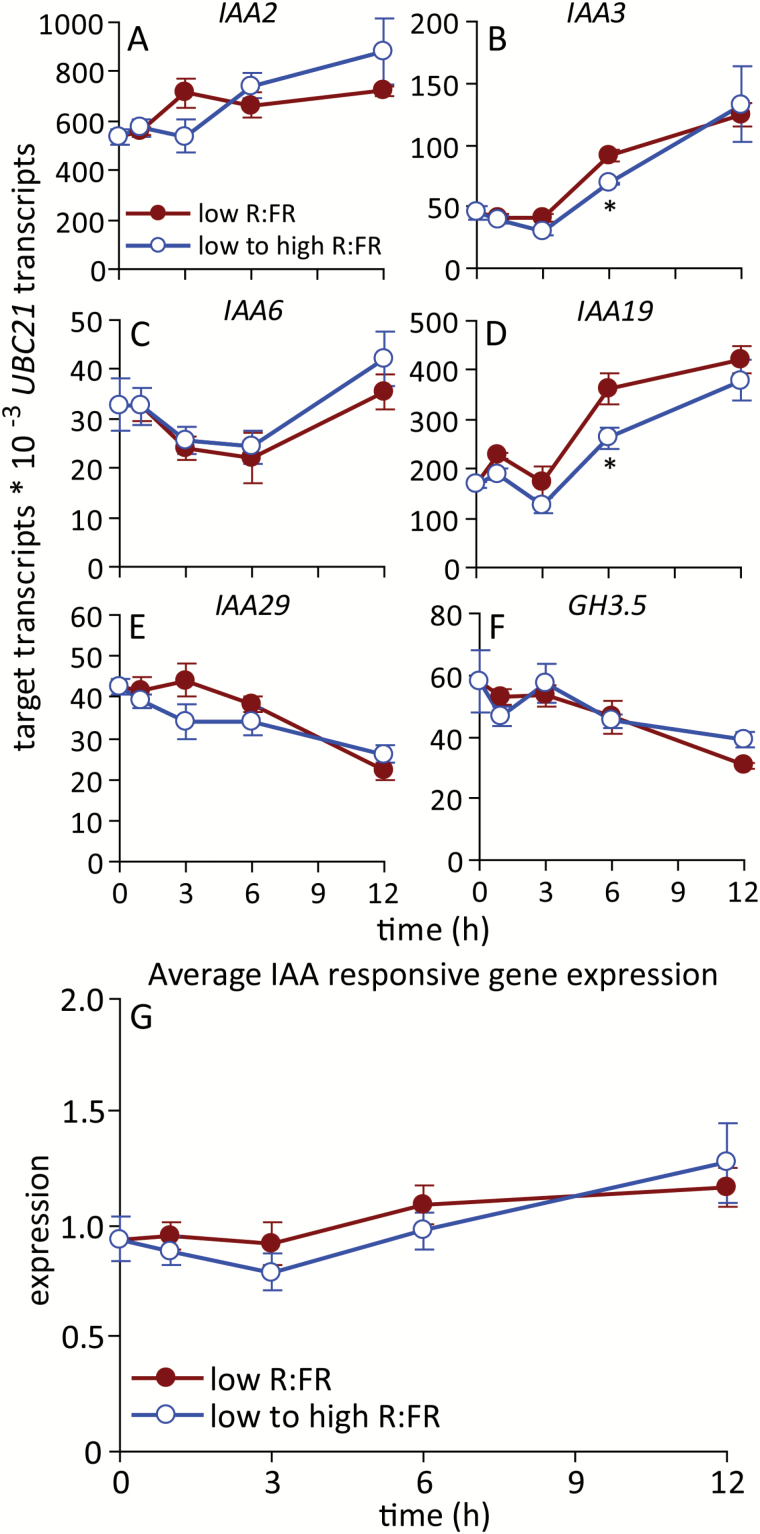
Expression of IAA-responsive genes in basal main stem segments under a low R:FR and at various times after increasing the R:FR. (**A**) *IAA2*, (**B**) *IAA3*, (**C**) *IAA6*, (**D**) *IAA19*, (**E**) *IAA29*, (**F**) *GH3.5*, (**G**) average positive normalized expression. Data are means ± SE with *n* = 4. Asterisks indicate a significant difference between a low and low to high R:FR at α = 0.05. This figure is available in colour at *JXB* online.

A low R:FR rapidly promotes the accumulation of IAA in young Arabidopsis seedlings (Tao *et al.*, 2008, Hornitschek *et al.*, 2012, Pacín *et al.*, 2016). Elevated systemic auxin signalling in the main stem, independent of IAA accumulation, was previously found to contribute to the suppression of branching in phyB-deficient Arabidopsis (Reddy and Finlayson, 2014). The role of systemic auxin in the response to an increased R:FR was evaluated to determine if this pathway contributes to the modulation of bud growth by the R:FR. IAA abundance in basal main stem segments decreased transiently in response to an elevated R:FR at 6 h, but recovered to levels observed in plants maintained under a low R:FR at 12 h (Fig. 7). Auxin signalling status was assessed using a panel of auxin-inducible genes (Goda *et al.*, 2008; Reddy and Finlayson, 2014), including *IAA2*, *IAA3*, *IAA6*, *IAA19*, *IAA29* and *GH3.5* (Fig. 8). Relative to a low R:FR, the expression of *IAA3* and *IAA19* was suppressed 6 h after increasing the R:FR, but there were no differences at any other times, or in the expression of the other four genes. The average expression of the auxin signalling panel was not altered by the R:FR (Fig. 8). Thus, while main stem IAA levels decreased transiently at 6 h, the molecular evidence indicated that there was only a brief and limited perturbation of specific auxin signalling components at 6 h.

### An increased R:FR promoted bud elongation more rapidly than decapitation

**Fig. 9.**
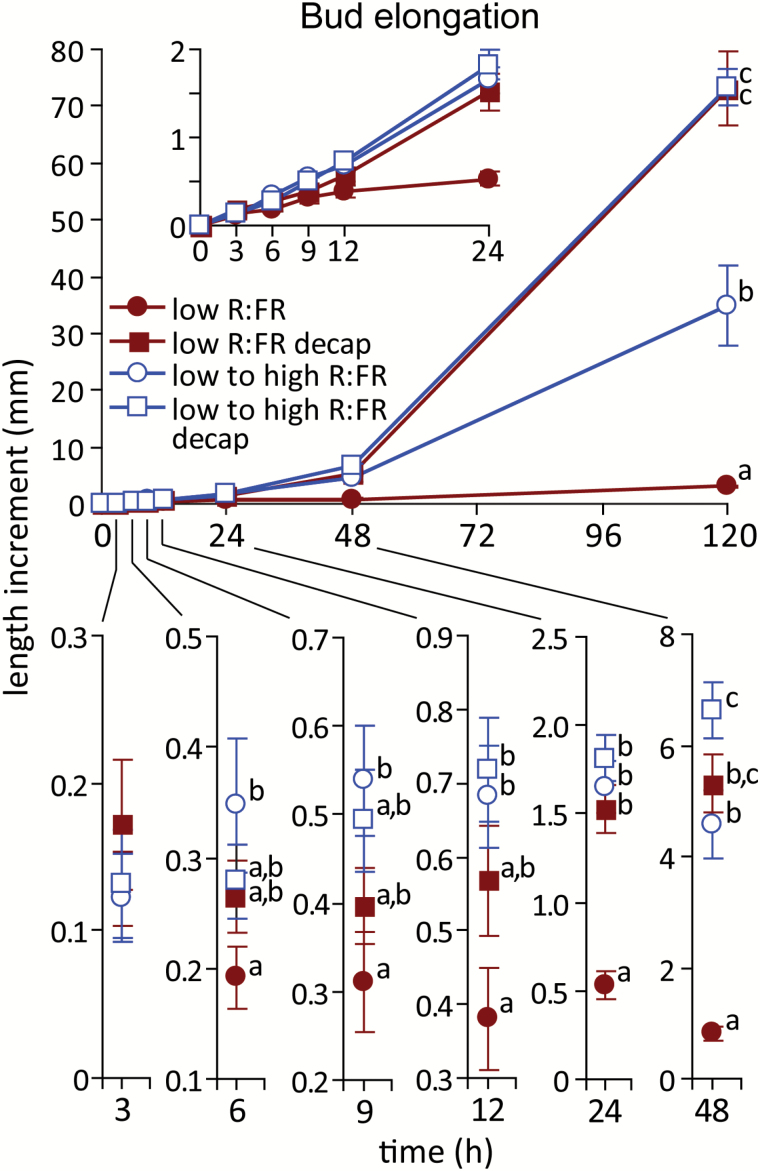
Elongation of bud n-2 under a low R:FR and at various times after decapitating the main stem and/or increasing the R:FR. Top panel shows full data set, inset magnifies the first 24 h, lower panel magnifies time points from 3 to 48 h. Data are means ± SE with *n* = 13. Values within time points with different letters are significantly different at α = 0.05. This figure is available in colour at *JXB* online.

In most species tested, including Arabidopsis, decapitation of the main shoot results in rapid growth of otherwise repressed buds. While both decapitation of the main stem and increasing the R:FR promote bud growth, information regarding potential commonalities, differences, and interactions between these treatments is lacking. These issues were explored by decapitating plants grown and maintained under a low R:FR, and comparing the timing and extent of bud growth with counterparts decapitated and provided with an increased R:FR. Buds were not significantly different in size at the start of treatments (low R:FR = 2.30 ± 0.20 mm, decapitated low R:FR = 2.05 ± 0.12 mm, low to high R:FR = 2.33 ± 0.16 mm, decapitated low to high R:FR = 2.51 ± 0.12 mm). In agreement with the data presented in Fig. 1 above, increasing the R:FR promoted bud growth within 6 h (Fig. 9). In contrast, decapitation of plants maintained under a low R:FR resulted in increased bud growth after a lag of 24 h. Furthermore, the combination of an increased R:FR and decapitation delayed the growth response by 6 h, compared to plants provided only with increased R:FR. While the effect of increased R:FR was more rapid than that produced by decapitation, decapitation resulted in stronger branch elongation by the end of the experiment (120 h), with both decapitation treatments generating branches that were over twice as long as those from plants only given an increased R:FR. The combination of an increased R:FR and decapitation did not promote final branch elongation more than decapitation alone.

## Discussion

### Rapid bud growth responses to an increased R:FR were preceded by the suppression of bud ABA accumulation and signalling relative to a low R:FR

The regulation of bud growth by R:FR signalling is a complex process involving at least two main pathways. Initial work indicated that an increased R:FR caused a reduction in bud-localized ABA, which allowed buds retarded by a low R:FR to grow more rapidly (Reddy *et al.*, 2013). These results were supported by independent research associating the expression of ABA-related genes with bud responses to the R:FR (González-Grandío *et al.*, 2013). A subsequent study demonstrated that the suppression of branching in phyB-deficient mutants resulted from systemic changes in auxin signalling in the main stem, independent of auxin abundance and transport (Reddy and Finlayson, 2014). Although the competition avoidance produced by a low R:FR may not exactly mirror the effects of phyB deficiency, the apparent similarities suggest that some of the effects of a low R:FR may also result from alterations in auxin abundance and/or signalling. In the current study, these two pathways were investigated in a time course analysis to determine how they might contribute to bud growth responses to the R:FR.

Bud growth was promoted very rapidly by increasing the R:FR. Both the abundance of ABA in buds and bud ABA signalling status decreased relative to a low R:FR prior (3 h post treatment) to the observed changes in bud growth. These changes may be directly related to bud function because ABA was necessary for the inhibition of lower bud elongation in a low R:FR (Reddy *et al.*, 2013), and exogenous ABA inhibited the growth of buds under a high R:FR (Yao and Finlayson, 2015). Furthermore, the expression of the cell cycle components *PCNA1* and *CYCA2;1* increased commensurate with, or after, the decrease in ABA, suggesting possible targets for ABA function, as previously described (Yao and Finlayson, 2015). These potential targets also included genes involved in auxin biosynthesis (*TAA1*) and transport (*PIN1*) within the bud itself. *TAA1* and *PIN1* showed similar expression responses to an increased R:FR, with significantly increased expression relative to a low R:FR at 6 h, as might be expected if the changes resulted from the measured decrease in ABA. This conclusion is complicated by the observation that both genes also exhibited increased expression at 1 h, followed by a decline at 3 h to the levels observed in buds from plants maintained under a low R:FR. Such a pattern of accumulation could indicate a rapid, transient, ABA-independent promotion of their expression by the R:FR, possibly priming the bud for eventual release by more sustained signals. Assessing the rapid effects of cytokinin application to buds maintained under a low R:FR or provided with a high R:FR for one to several hours could help resolve the role of the initial transient increase in *TAA1* and *PIN1* expression.

### Main stem IAA accumulation and signalling responses to an increased R:FR did not correlate with rapid bud growth responses

An increased R:FR also altered the abundance of IAA in the main stem, and impacted the expression of two of the six auxin signalling components surveyed. However, both phenomena were only transiently detected at 6 h, and thus occurred later than the changes observed in bud ABA physiology and at the same time that changes in bud elongation were measured. Decapitation was employed to determine the response of buds to complete removal of the apical auxin source. While increased bud elongation was observed 6 h after increasing the R:FR, decapitation promoted growth in plants maintained under a low R:FR only by 24 h after dissection. This severe and abrupt treatment would be expected to impact auxin levels and signalling in less than the 6 h required for the observed weak, transient auxin response to an increased R:FR, but decapitation still required more time to manifest in increased bud elongation than the light treatment. The combination of the delayed main stem auxin response to an altered R:FR and the delayed growth response to auxin depletion by decapitation indicate that auxin is unlikely to be the primary signal for bud release under an increased R:FR. Conversely, the long-term effects of decapitation were considerably stronger than those associated with an increased R:FR, and decapitation eventually overcame the low R:FR inhibition of bud elongation completely, indicating that R:FR signal perception and transduction events generated by the shoot apex or stem are necessary for persistent inhibition of bud growth. Thus, the primary early effects of an increased R:FR on bud growth can be attributed to alterations in bud ABA physiology, whereas later elongation responses may also involve altered systemic auxin physiology.

The decline in bud ABA abundance following exposure to a high R:FR occurred with a timing similar to changes in bud hormone status following decapitation in pea (*Pisum sativum*) and chickpea (*Cicer arietinum*). Decapitation promoted bud outgrowth in pea in 4–6 h (Morris *et al.*, 2005). Decapitation also increased cytokinin abundance in pea buds within 3 h (Tanaka *et al.*, 2006) and the buds of excised pea shoot sections showed an increased ability to transport auxin 2 h after removing the terminal bud (Balla *et al.*, 2011). Rapid hormonal responses to decapitation were also observed in buds of chickpea, with cytokinin abundances increasing within 4 h, while ABA levels declined within 1 h (Mader *et al.*, 2003). The delayed onset of bud outgrowth in Arabidopsis following decapitation compared to pea may reflect either an intrinsically slower response, or may result from the prior growth of these plants under a low R:FR.

It was interesting to note that the combination of decapitation and an increased R:FR promoted bud elongation less rapidly than increasing the R:FR alone, suggesting that the main stem may provide a positive regulator of early bud growth necessary for the rapid response. Alternatively, decapitation might differentially stimulate the growth of buds n and/or n-1, which could result in increased correlative inhibition of bud n-2 via the auxin transport competition theory (Bennett *et al.*, 2006; Prusinkiewicz *et al.*, 2009; Bennett *et al.*, 2016). However, at the termination of the experiment, the combination of a high R:FR and decapitation did not significantly increase the length of the upper branches compared to a high R:FR alone (see Supplementary Fig. 2 at *JXB* online), making this seem unlikely.

A previous transcriptome profiling study using the same experimental system but only a single 3 h time point provided evidence that cytokinin signalling was upregulated in response to exposure to a high R:FR (Reddy *et al.*, 2013). Gene ontology (GO) terms associated with cytokinin response were overrepresented and the expression of cytokinin-responsive type A *RESPONSE REGULATOR* (*ARR*) genes, including *ARR4*, *ARR5*, *ARR6*, *ARR7*, and *ARR15*, were induced from about 1.7 to 6-fold 3 h after increasing the R:FR. However, expression of the cytokinin biosynthesis *ISOPENTENYLTRANSFERASE* genes was not altered. GO analysis did not indicate overrepresentation of terms associated with the MORE AXILLARY BRANCHING (MAX) pathway, but *MAX2* expression declined about 1.5-fold following exposure to a high R:FR. Thus, other hormonal pathways besides ABA and auxin are probably also rapidly modulated by the R:FR; defining the timing and how they interact is an objective for future research.

### Bud *BRC1* expression and ABA physiology were dynamically expressed

The abundance of ABA in buds maintained under a low R:FR was not static, but varied with time, reaching a maximum at 6 h (7 h after dawn). The expression of ABA-responsive genes and *BRC1* in buds under a low R:FR was also dynamic and increased through the early part of the day. Even in a low R:FR, bud n-2 showed some elongation in spite of the increased expression of these negative regulators of bud growth, indicating that these factors are insufficient for complete arrest. As discussed previously (Yao and Finlayson, 2015), Arabidopsis buds do not appear to exhibit true dormancy, as in all cases where observations have been made, buds that superficially appeared dormant in fact showed some measurable growth (Finlayson *et al.*, 2010, Su *et al.*, 2011, Reddy *et al.*, 2013). In the present study, bud growth in plants provided with a high R:FR may not be associated with an absolute reduction in the expression/accumulation of these negative regulators, but rather a lack of increase.

The apparent increase in expression/accumulation patterns of *BRC1* and ABA may indicate that the bud growth response is gated to permit the process to initiate at a particular time of the day, presumably in the morning. There is considerable support for clock gating of ABA signalling (see Seung *et al.*, 2012); however, less information is available regarding potential *BRC1* expression rhythms. BRC1 is a member of the TCP protein family. Most TCP family genes show diurnal expression patterns (Giraud *et al.*, 2010), although rhythmic expression of *BRC1* was not tested due to its low abundance. Because ABA accumulation in lower buds is dependent on BRC1 function (Yao and Finlayson, 2015), it is tempting to speculate that increased diurnal expression of *BRC1* results in the accumulation of ABA, which contributes to suppression of bud growth later in the day. Therefore, *BRC1* expression could be the gated factor inhibiting bud growth, rather than, or perhaps in addition to, ABA signalling. Because the diurnal bud growth response was not assessed, this hypothesis currently remains untested.

### Does the axillary bud autonomously sense and respond to the R:FR?

While BRC1 is necessary for maintaining elevated levels of ABA in lower buds (Yao and Finlayson, 2015), it is not known how ABA levels and signalling are suppressed by a high R:FR. The rapid modulation of ABA homeostasis by an increased R:FR could indicate that the bud itself is the primary site of perception and signal transduction for this response. In this scenario, changes in the R:FR detected by phytochromes within bud cells would potentially be transduced by PHYTOCHROME INTERACTING FACTORs (PIFs) to alter the expression of ABA biosynthesis and/or metabolism genes, either through PIF-mediated changes in *BRC1* expression, or more directly. Phytochrome action was shown to suppress ABA accumulation in *Lemna gibba* (Weatherwax *et al.*, 1996) and ABA accumulation in Arabidopsis seeds was suppressed by phyB regulation of both its biosynthesis and catabolism (Seo *et al.*, 2006). The key ABA biosynthetic gene *NINE-CIS-EPOXYCAROTENOID DIOXYGENASE 3* (*NCED3*) and the ABA hydroxylase gene *CYTOCHROME P450*, *FAMILY 707*, *SUBFAMILY A*, *POLYPEPTIDE 4* (*CYP707A4*) were found to be regulated by PIF4/PIF5, which contribute to responses to the R:FR (Hornitschek *et al.*, 2012). Additionally, *CYP707A4* and its homolog *CYP707A3*, the *NCED3* homolog *NCED4*, and genes encoding the ABA signalling transcription factors *ABI5* and *ABSCISIC ACID RESPONSIVE ELEMENTS-BINDING FACTOR 3* were also identified as binding targets for PIF5 (Hornitschek *et al.*, 2012). Investigating potential links between canonical phyB signal transduction via PIFs and the expression of ABA biosynthesis, catabolism, and signalling genes in axillary buds may provide critical data to better understand the regulation of bud growth by the R:FR.

## Supplementary Data

Supplementary data are available at *JXB* online.

Supplementary Fig. 1. Spectra of the light sources used for plant growth.

Supplementary Fig. 2. Lengths of upper rosette branches (n and n-1) of plants grown under a low R:FR, then provided with a high R:FR or maintained under a low R:FR, with and without decapitation at 120 h after initiating treatments.

## Supplementary Material

supplementary_figures_S1_S2

## Abbreviations:

ABA: abscisic acid
ARR: *RESPONSE REGULATOR*
BRC1: *BRANCHED 1*
CYCA2;1: *CYCLIN A2;1*
CYP707A4: *CYTOCHROME P450, FAMILY 707, SUBFAMILY A, POLYPEPTIDE 4*
GO: gene ontology
HIS1-3: *HISTONE H1-3*
IAA: indole-3-acetic acid
IAA2: *INDOLE-3-ACETIC ACID INDUCIBLE 2*
MAX: MORE AXILLARY BRANCHING
NAP: *ARABIDOPSIS NAC DOMAIN CONTAINING PROTEIN 29*
NCED: *NINE-CIS-EPOXYCAROTENOID DIOXYGENASE*
PCNA1: *PROLIFERATING CELL NUCLEAR ANTIGEN 1*
PIF: PHYTOCHROME INTERACTING FACTOR
phy: phytochrome
PIN1: *PIN-FORMED 1*
PP2-A5: *PHLOEM PROTEIN 2 A5*
RAP2.6: *RELATED TO AP2 6*
R:FR: red light to far red light ratio
TAA1: *TRYPTOPHAN AMINOTRANSFERASE OF ARABIDOPSIS 1*
TCP: TEOSINTE BRANCHED1/CYCLOIDEA/PCF.
