# Supplementary material for: The ratio of red light to far red light alters Arabidopsis axillary bud growth and abscisic acid signalling before stem auxin changes
Source: J Exp Bot. 2017 Jan 6;68(5):943–52. doi: 10.1093/jxb/erw479 (PMC5444464; doi:10.1093/jxb/erw479)
Supplement: supplementary_figures_S1_S2 [file erw479_suppl_supplementary_figures_S1_S2.pdf]

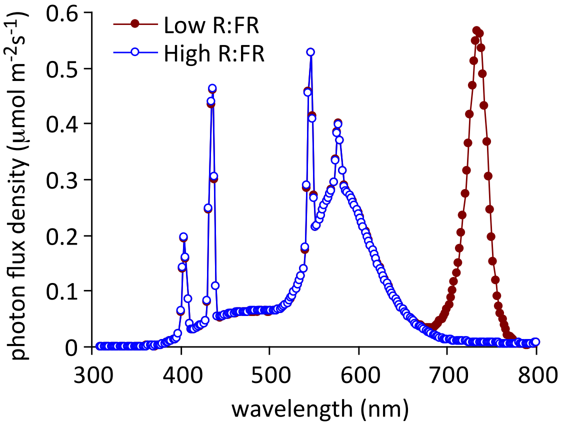

Supplemental figure 1. Spectra of the light sources used for plant growth.

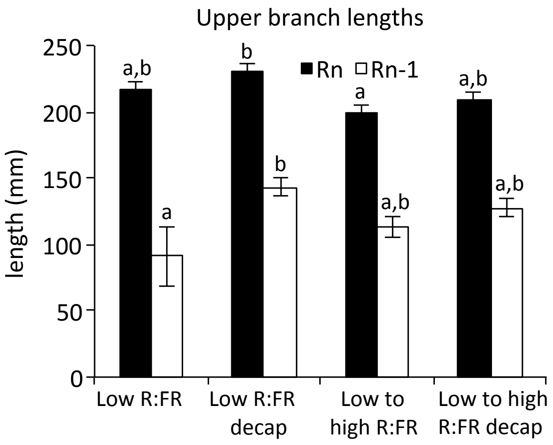

Supplemental figure 2. Lengths of upper rosette branches (n and n-1) of plants grown under low R:FR, then provided with high R:FR or maintained under low R:FR, with and without decapitation at 120 h after initiating treatments. Data are means ± SE with n = 13. Bars with different letters are significantly different at $\alpha = 0.05$.
